# Inflow restrictions can prevent epidemics when contact tracing efforts are effective but have limited capacity

**DOI:** 10.1098/rsif.2020.0351

**Published:** 2020-09-09

**Authors:** Hannes Malmberg, Tom Britton

**Affiliations:** 1 Department of Economics, University of Minnesota, Minneapolis, MN 55455, USA; 2 Department of Mathematics, Stockholm University, Sweden

**Keywords:** epidemics, travel restrictions, contact tracing

## Abstract

When a region tries to prevent an outbreak of an epidemic, two broad strategies are available: limiting the inflow of infected cases by using travel restrictions and quarantines or limiting the risk of local transmission from imported cases by using contact tracing and other community interventions. A number of papers have used epidemiological models to argue that inflow restrictions are unlikely to be effective. We simulate a simple epidemiological model to show that this conclusion changes if containment efforts such as contact tracing have limited capacity. In particular, our results show that moderate travel restrictions can lead to large reductions in the probability of an epidemic when contact tracing is effective but the contact tracing system is close to being overwhelmed.

## 1. Introduction

Two main factors determine if, and when, a region will be affected by an epidemic like the current COVID-19 outbreak, be it the first epidemic or a second wave after a successful lockdown has eliminated internal spread. The first factor is the rate *λ* at which infectious individuals (either visitors or returning local residents) enter the country. The second factor is the probability *π* that such an entry gives rise to a major outbreak.^1^

Potential preventive measures by health authorities can target reductions of *λ* using, for example, travel restrictions or quarantines, or reductions of *π* using, for example, contact tracing in conjunction with the isolation of imported cases (henceforth ‘contact tracing'). Quite often, preventive measures aim at reducing both *λ* and *π*.

Contact tracing can be fully effective (i.e. *π* = 0) if it manages to bring the epidemic's effective reproduction number *R* below 1 during the early stage of the outbreak, where *R* is defined as the expected number of infections caused by an infected individual. Intuitively, if each individual, on average, infects less than one other individual, a large outbreak is not possible.

We use a simple epidemiological model to analyse the effects of reducing *π* and *λ* on epidemic outbreaks. The background to our work is a literature that has found small effects from regulating the inflow rate *λ*. Anzai *et al*. [1] show that inflow reductions alone (assuming *π* > 0) cannot prevent an epidemic outbreak from taking place and at best delay epidemic onset, often for just a very limited time. Chinazzi *et al*. [2] study the effect of reducing the inflow of infected individuals while simultaneously reducing *π* in the community at large and also find that inflow reductions can only marginally delay an epidemic unless *π* is reduced drastically. These pessimistic findings mirror those from a large number of earlier models [3–8]. The World Health Organization's 2014 Systematic Review on the role of travel restrictions in containing pandemic influenza reviewed 23 papers and concluded that a 90% reduction in international air travel would only slow down a pandemic by 3–4 weeks and would not prevent the introduction of a pandemic into any given country [9].

We focus on the joint effect of inflow reductions and contact tracing. We incorporate contact tracing by having two different outbreak probabilities: one negligible for incoming cases that are contact traced effectively and another much higher probability for incoming cases that are not contact traced. With no capacity constraints, changing *λ* does not meaningfully affect the probability of an outbreak, regardless of whether effective contact tracing is in place or not. If effective contact tracing is not in place, reducing the inflow rate *λ* will only marginally delay the epidemic, in line with the literature findings above. If effective contact tracing is in place, there will not be an epidemic for any *λ*, so reductions in *λ* are again irrelevant.

However, this conclusion relies critically on the assumption of unlimited capacity in contact tracing, i.e. that all imported cases are contact traced effectively irrespective of how many are currently being traced. We show that when contact tracing is effective but has limited capacity, reducing *λ* may well be very effective in reducing the risk of an outbreak. Regulating *λ* is particularly important when the contact tracing system is close to being overwhelmed by new cases arriving from elsewhere, in which case even moderate reductions in *λ* can strongly reduce the probability of an epidemic outbreak.

Since contact tracing is both resource and labour intensive, we believe that our limited capacity assumption is reasonable. We also conjecture that, while our model is simple, our qualitative findings translate to more realistic set-ups. Hence, we think that epidemiological models used for policy analysis should incorporate capacity constraints, since they might otherwise underestimate the potential of travel restrictions to prevent epidemic outbreaks (or the re-emergence of an epidemic).

## 2. Model

### 2.1. Set-up

Formally, we study a stochastic epidemic model where infected cases arrive at some Poisson rate *λ*. Without contact tracing, each new case leads to an epidemic outbreak with some probability *π_NT_* > 0. The containment effort consists of contact tracing that reduces the probability of an epidemic outbreak to *π_T_* ≥ 0. We say that contact tracing is *effective* if *π_T_* = 0 and is *imperfect* if *π_T_* > 0.

To model potential capacity constraints, we assume that contact tracing is conducted by a set of *n* teams which process every case arrival with an intensity *μ* (so the mean time for completing contact tracing is 1/*μ*). If a case arrives when at least one team is free, that case has probability *π_T_* of leading to an epidemic. If all teams are occupied when an infected case arrives, that case is lost to the system and causes an epidemic with probability *π_NT_*. We say that contact tracing has *unlimited capacity* if *n* = ∞ and has *limited capacity* if *n* < ∞.

**Figure 1.**
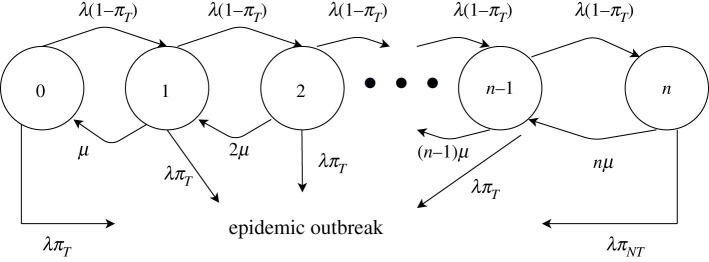
Simplified model of an epidemic outbreak with contact tracing. This diagram outlines the basic evolution of a disease from emergence to epidemic outbreak in the presence of a contact tracing system. When the system is imperfect, each traced case has a positive probability of leading to an epidemic, regardless of the arrival rate of new cases, the rate at which cases are processed or the number of cases that can be processed at once. When the system is effective, an outbreak will only occur if the system's capacity is limited and not all newly arriving cases can be processed.

This set-up can be modelled as a queuing system where the state is the number of people currently in the contact tracing system. To analyse the probability of an epidemic outbreak at different time horizons, we add the outbreak as an additional, absorbing state. The set-up is illustrated in figure 1.

### 2.2. The effect of inflow reductions on the outbreak probability

With this set-up, we consider the effect of varying *λ* under four different combinations of parameters: contact tracing being imperfect versus effective and having unlimited versus limited capacity. Doing this, we confirm the recent literature's main finding: varying *λ* is relatively inconsequential when capacity is unlimited. However, we also find that varying *λ* can be very important when contact tracing is effective but has limited capacity. We proceed by discussing each case in turn.

#### 2.2.1. Case 1—imperfect contract tracing with unlimited capacity

In this case, there is an epidemic outbreak at a constant rate *λπ_T_* > 0. At a horizon *t*, the probability of an outbreak is

$$p_{\mathrm{OB}}(t)={1-e}^{-\lambda *\pi_{t}*t}.$$

Reducing *λ* can proportionally delay the outbreak but cannot stop it.

#### 2.2.2. Case 2—effective contact tracing with unlimited capacity

In this case, all arriving cases have zero probability of causing an epidemic. Thus, regardless of *λ*, there will not be an outbreak.

#### 2.2.3. Case 3—imperfect contact tracing with limited capacity

In this case, epidemics break out at a rate *λπ_T_* when the contact tracing system is below capacity and at a rate *λπ_NT_* > *λπ_T_* when at capacity. By contrast with case 1, reducing *λ* has the benefit of reducing the probability of being at full capacity. However, since the containment system is not fully effective, reducing *λ* can still only delay the outbreak.

#### 2.2.4. Case 4—effective contact tracing with limited capacity

In this case, epidemics do not occur when the contact tracing system is below capacity but do occur at a rate *λπ_NT_* > 0 when at capacity. Thus, by preventing the system from reaching full capacity, reducing *λ* can be very effective at preventing outbreaks. The effect of *λ* can also be highly nonlinear. Indeed, unless the queue was truncated at *n*, the value *λ* = *nμ* would be a critical value where the system would change discretely from being a subcritical to a supercritical system.

## 3. Simulation

To illustrate our findings, we perform simulations varying *λ* under each of these four different cases. All simulation parameters are given in table 1. We assume that, when capacity is limited, there are *n* = 100 tracing teams and cases are handled by a team, on average, in 2 days (i.e. *μ* = 0.5). We consider three infected case arrival rates: a baseline rate *λ* = 80, a moderate reduction *λ* = 40 and a strong reduction *λ* = 20.^2^ When contact tracing is imperfect we assume that *π_T_* = 0.1%, and when contact tracing is effective we assume that *π_T_* = 0.001%.^3^ In the absence of any contact tracing, we assume that *π_NT_* = 10%.^4^

**Table 1.** All parameter values used in our model-simulated experiments. For parameters where multiple values are considered, the text in parentheses indicates each value's relevant case.

parameter	value(s)
*n*	100 (*limited*), ∞ (*unlimited*)
*μ*	0.5
*λ*	80 (*baseline*), 40 (*moderate*), 20 (*strong*)
*π_T_*	10^−3^ (*imperfect*), 10^−5^ (*effective*)
*π_NT_*	0.1

**Figure 2.**
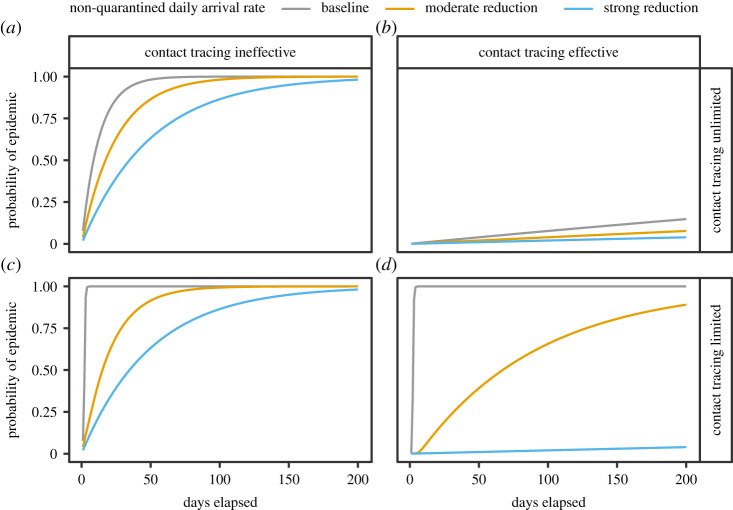
Model-simulated probability of an epidemic outbreak as a function of time. Each panel shows the results of model simulations estimating the probability of an epidemic occurring as a function of time given one of three possible arrival rates for new infected cases: a baseline arrival rate, a moderately reduced arrival rate and a strongly reduced arrival rate. Here (*a*,*b*) display results from simulations where contact tracing is assumed to have unlimited capacity, while (*c*,*d*) assume limited capacity. Here (*a*,*c*) display results from simulations where contract tracing is assumed to be less than fully effective, while (*b*,*d*) assume that it is fully effective.

The results for each case are displayed in figure 2, with the number of days on the horizontal axis and the probability of an epidemic outbreak on the vertical axis. The first row shows the first two cases, where contact tracing has unlimited capacity. In the left-hand panel, contact tracing is imperfect, and reducing *λ* only delays the outbreak. In the right-hand panel, contact tracing is effective and there is a low probability of an outbreak independent of *λ*.

The second row shows the two cases where contract tracing has limited capacity. In the left-hand panel, contact tracing is imperfect and the result is qualitatively similar to the case with unlimited capacity; reducing *λ* only delays the epidemic, with a somewhat stronger effect in that the epidemic starts immediately in the absence of inflow restrictions. By contrast, when contact tracing is effective, the result is very different from that with unlimited capacity. This case is shown in the right-hand panel, where reducing *λ* can change the probability of an epidemic from virtual certainty to almost nil by preventing the tracing system from being overwhelmed.

## 4. Limitations

The model makes a simplifying assumption that contact tracing resources are only used for imported cases. In practice, domestic cases also have to be contact traced, since every imported case will not be isolated before it causes secondary transmissions. In such a situation, contact tracing will be effective if it brings down the effective reproduction number below 1, in which case standard branching process logic implies that the epidemic dies out before it ever becomes large.

We conjecture that our qualitative conclusions are not affected by allowing for domestic spread. Just as before, travel restrictions are not important with unlimited contact tracing: either contact tracing is ineffective, in which case travel restrictions can only slow down the epidemic, or contact tracing is effective, in which case there will not be an epidemic regardless of travel restrictions. By contrast, if contact tracing is effective but has limited capacity, there can still be an epidemic because of an overload of the system, and travel restrictions will affect the probability that such an overload happens.

The main difference from introducing domestic spread is that a larger set of parameters becomes relevant for policy. For example, the probability of being overloaded now will depend on how quickly imported cases are discovered, how infectious they are during the waiting time, how much they interact during the waiting time and how many infections each case causes on average, and also on factors such as the dispersion in the number of infections per case and dispersion in the time taken to process a case.

We also assume that the capacity of the system is fixed over time. However, during the COVID-19 pandemic, many countries have invested in expanded contact tracing capacity as the epidemic has progressed. An interesting extension to the model would be to allow for a gradual expansion of the contact tracing system. In this situation, it is likely that travel restrictions could adapt dynamically over time to become looser as the contact tracing system achieves a larger capacity.

## 5. Conclusion

Taking stock, we conclude that introducing capacity constraints can imply large changes in the effectiveness of inflow restrictions. Instead of inflow restrictions leading to a gradual delay of an epidemic, there are nonlinear effects once the system goes below capacity. Thus, in cases where systems are at the risk of being overrun, even moderate travel restrictions can be highly effective in reducing the risk for a local epidemic.

While most rich countries are now beyond the point of preventing domestic outbreaks of COVID-19, we believe that the reasoning in this paper is still relevant for countries that have outbreaks that are later brought under control. In these cases, inflow restrictions may be helpful in preventing an epidemic from re-emerging, as they allow the country to stay below capacity in their contact tracing efforts.

## Endnotes

1 Our analysis focuses on the probability of an epidemic outbreak. We do not study the subsequent evolution of the disease after this event. In general, inflow restrictions become inconsequential once the amount of local transmission is much larger than the number of imported cases.

2 These rates are chosen to pass the critical value of *nμ* = 50, the maximum number of cases the teams can handle per day.

3 We use a strictly positive value in order to capture how even a contact tracing system that generally reduces *R* below 1 can still miss an infection chain by a stroke of bad luck.

4 The parameters are not meant to be realistic, but to illustrate qualitative properties of the model. In practice, a country could estimate *λ* by analysing the number of incoming travellers, the prevalence of disease at sending locations and the overrepresentation of disease among travellers, whereas *μ* could be approximated from information about average labour requirements in resolving a contact tracing case. The terms *π_T_* and *π_NT_* would have to be inferred from a richer epidemiological model.

## Data accessibility

Replication code for the simulation exercises is available upon request.

## References

[1] Anzai A et al. 2020 Assessing the impact of reduced travel on exportation dynamics of novel coronavirus infection (COVID-19). J. Clin. Med. 9, 601 (10.3390/jcm9020601) PMC7073579 32102279

[2] Chinazzi M et al. 2020 The effect of travel restrictions on the spread of the 2019 novel coronavirus (COVID-19) outbreak. Science 368, 395–400. (10.1126/science.aba9757) 32144116 PMC7164386

[3] Mateus ALP, Otete HE, Beck CR, Dolan GP, Nguyen-Van-Tam JS 2014 Effectiveness of travel restrictions in the rapid containment of human influenza: a systematic review. Bull. World Health Organ. 92, 868–880D. (10.2471/BLT.14.135590) 25552771 PMC4264390

[4] Lam EHY, Cowling BJ, Cook AR, Wong JYT, Lau MSY, Nishiura H 2011 The feasibility of age-specific travel restrictions during influenza pandemics. Theor. Biol. Med. Model. 8, 44 (10.1186/1742-4682-8-44) 22078655 PMC3278369

[5] Bajardi P, Poletto C, Ramasco JJ, Tizzoni M, Colizza V, Vespignani A 2011 Human mobility networks, travel restrictions, and the global spread of 2009 H1N1 pandemic. PLoS ONE 6, e16591 (10.1371/journal.pone.0016591) 21304943 PMC3031602

[6] Degli Atti ML, Merler S, Rizzo C, Ajelli M, Massari M, Manfredi P, Furlanello C, Tomba GS, Iannelli M 2008 Mitigation measures for pandemic influenza in Italy: an individual based model considering different scenarios. PLoS ONE 3, e1790 (10.1371/journal.pone.0001790) 18335060 PMC2258437

[7] Cooper BS, Pitman RJ, Edmunds WJ, Gay NJ 2006 Delaying the international spread of pandemic influenza. PLoS Med. 3, e212 (10.1371/journal.pmed.0030212) 16640458 PMC1450020

[8] Epstein JM, Goedecke DM, Yu F, Morris RJ, Wagener DK, Bobashev GV 2007 Controlling pandemic flu: the value of international air travel restrictions. PLoS ONE 2, e401 (10.1371/journal.pone.0000401) 17476323 PMC1855004

[9] Eichner M, Schwehm M, Wilson N, Baker MG 2009 Small islands and pandemic influenza: potential benefits and limitations of travel volume reduction as a border control measure. BMC Infect. Dis. 9, 160 (10.1186/1471-2334-9-160) 19788751 PMC2761921

